# Supplementary material for: Stress management strategies and controlling emotion among Polish nurses and midwives who suffer COVID: cross-sectional study
Source: PeerJ. 2025 Sep 22;13:e19816. doi: 10.7717/peerj.19816 (PMC12462688; doi:10.7717/peerj.19816)
Supplement: Supplemental Information 2 [file peerj-13-19816-s002.docx]

Legend:

Sex/gender:

K - women

M – mean

Place of work:

1 -hospital

2 - clinic

Hospital's degree of reference:

0 - no information

I - I degree of reference

II - 2^nd^ degree of reference

III - 3^rd^ degree of reference

Another place:

0 - no information

Degree of reference:

0 - no information

Place of residence:

1 - Provincial City

2– City

3 – Village

COVID-19 pre-infection health assessment:

1 – Bad

2 – Average

3 – Moderate

4 – Good

5 – Very good

course of the disease (Covid-19):

1 - Asymptomatic

2 - Mild

3 – Heavy

Test with which Sars-CoV-2 infection was confirmed:

1 – Serological

2 – Genetic

3 – Antigen

4 - The test was not performed

Symptoms of Covid-19: Cough; Fever; Shortness of breath/ difficulty breathing; Olfactory disorders; Taste disorders; Loss of appetite; Diarrhea; Shivers; Myalgia (muscle pain); Nausea and vomiting; Headache; Sore throat; Conjunctivitis; Irritability; Confusion; Memory problems; Skin rash; Discoloration of fingers and toes; Anxiety; Depressive states; Sleep disorders and Neurological disorders

1 – yes

0 – no

Hospitalization due to covid-19:

1 – yes

0 – no

Complications of COVID-19: Fatigue; Shortness of breath; Cough; Joint pains; Chest pain; Lower mood; Muscle pains; Headaches; Dizziness; Recurrent fever; Heart palpitations; Loss of smell; Taste disorders; Short-term memory loss; Difficulties with concentration; More frequent infections

1 – yes

0 – no

Coping Inventory for Stressful Situations (CISS) (CISS1 – CISS 48)

1 - means never,

2 - very rarely,

3 - sometimes,

4 - often,

5 - very often

The questionnaire consists of 48 statements rated on a five-point Likert scale, which ranks scores in 3 styles of coping with stress: Task-Oriented Coping (TOC), Emotion-Oriented Coping (EOC) and Avoidance-Oriented Coping (AOC) in two variations: Distraction (D subscale) and Social Diversion (SD subscale).

48 statements in appendix Coping Inventory for Stressful Situations (CISS) - key

Courtauld Emotional Control Scale (CECS).

Emotional control was measured using the Courtauld Emotional Control Scale (CECS), adapted in Poland by Juczynski (2009) developed by Watson and Greer (Juczyński, 2012). It consists of 21 statements on the disclosure of three basic emotions, i.e. anger, depression and anxiety.

Expressing consent to use the tool (CECS and CISS) is equivalent to purchasing the tool in the Psychological Testing Workshop of the Polish Psychological Association (Attachments: invoices 1 036/FIS/2016/02298 and 1 393/FIS/2021/01665).
