# Supplementary material for: Stress management strategies and controlling emotion among Polish nurses and midwives who suffer COVID: cross-sectional study
Source: PeerJ. 2025 Sep 22;13:e19816. doi: 10.7717/peerj.19816 (PMC12462688; doi:10.7717/peerj.19816)
Supplement: Supplemental Information 3 [file peerj-13-19816-s003.pdf]

Coping Inventory for Stressful Situations (CISS)

CISS was founded by Norman S. Endler and James D. Parker

Instructions: The following are ways people react to various difficult, stressful, or upsetting situations. Please circle a number from 1 to 5 for each item. Indicate how much you engage in these types of activities when you encounter a difficult, stressful, or upsetting situation.

			Scales					
ITEM	Coping Mechanism	Category	Not All				Very Much	FILL IN YOUR ANSWER HERE
1	Schedule my time better	TO	1	2	3	4	5	
2	Focus on the problem and see how I can solve it.	TO	1	2	3	4	5	
3	Think about the good times I've had	AO	1	2	3	4	5	
4	Try to be with other people.	AO	1	2	3	4	5	
5	Blame myself for procrastinating	EO	1	2	3	4	5	
6	Do what I think is best	TO	1	2	3	4	5	
7	Become preoccupied with aches and pains.	EO	1	2	3	4	5	
8	Blame myself for having gotten into this situation.	EO	1	2	3	4	5	
9	Window shop.	AO	1	2	3	4	5	
10	Outline my priorities.	TO	1	2	3	4	5	
11	Try to go to sleep.	AO	1	2	3	4	5	
12	Treat myself to a favorite food or snack.	AO	1	2	3	4	5	
13	Feel anxious about not being able to cope.	EO	1	2	3	4	5	
14	Become very tense.	EO	1	2	3	4	5	
15	Think about how I solved similar problems.	TO	1	2	3	4	5	
16	Tell myself that it is really not happening to me.	EO	1	2	3	4	5	
17	Blame myself for being too emotional about the situation.	EO	1	2	3	4	5	
18	Go out for a snack or meal.	AO	1	2	3	4	5	
19	Become very' upset.	EO	1	2	3	4	5	
20	Buy myself something.	AO	1	2	3	4	5	
21	Determine a course of action and follow it.	TO	1	2	3	4	5	
22	Blame myself for not knowing what to do.	EO	1	2	3	4	5	

23	Go to a party.	AO	1	2	3	4	5	
24	Work to understand the situation.	TO	1	2	3	4	5	
25	"Freeze" and not know what to do	EO	1	2	3	4	5	
26	Take corrective action immediately.	TO	1	2	3	4	5	
27	Think about the event and learn from my mistakes	TO	1	2	3	4	5	
28	Wish that I could change what had happened or how I felt.	EO	1	2	3	4	5	
29	Visit a friend.	AO	1	2	3	4	5	
30	Worry about what I am going to do.	EO	1	2	3	4	5	
31	I spend time with a special person.	AO	1	2	3	4	5	
32	Go for a walk.	AO	1	2	3	4	5	
33	Tell myself that it will never happen again.	EO	1	2	3	4	5	
34	Focus on my general inadequacy	EO	1	2	3	4	5	
35	Talk to someone whose advice I value.	AO	1	2	3	4	5	
36	Analyze the problem before reacting.	TO	1	2	3	4	5	
37	Phone a friend.	AO	1	2	3	4	5	
38	Get angry.	EO	1	2	3	4	5	
39	Adjust my priorities	TO	1	2	3	4	5	
40	See a movie.	AO	1	2	3	4	5	
41	Get control of the situation.	TO	1	2	3	4	5	
42	Make efforts to get things done.	TO	1	2	3	4	5	
43	Come up with several different solutions to the problem.	TO	1	2	3	4	5	
44	Take some time off and get away from the situation. 3	AO	1	2	3	4	5	
45	Take it out on other people.	EO	1	2	3	4	5	
46	Use the situation to prove that I can do it.	TO	1	2	3	4	5	
47	Try' to be organized so I can be on top of the situation.	TO	1	2	3	4	5	
48	Watch TV.	AO	1	2	3	4	5	

Task Oriented	0
Emotional Oriented	0
Avoidance Oriented	0

Item No.	Coping Mechanism	Category	Scales				
			Not at All				Very Much
1	Take some time off and get away from the situation	AO	1	2	3	4	5
2	Focus on the problem and see how I can solve it	TO	1	2	3	4	5
3	Blame myself for having gotten into this situation	EO	1	2	3	4	5
4	Treat myself to a favorite food or snack	AO	1	2	3	4	5
5	Feel anxious about not being able to cope	EO	1	2	3	4	5
6	Think about how I solved similar problems	TO	1	2	3	4	5
7	Visit a friend	AO	1	2	3	4	5
8	Determine a course of action and follow it	TO	1	2	3	4	5
9	Buy myself something	AO	1	2	3	4	5
10	Blame myself for being too emotional about the situation	EO	1	2	3	4	5
11	Work to understand the situation	TO	1	2	3	4	5
12	Become very upset	EO	1	2	3	4	5
13	Take corrective action immediately	TO	1	2	3	4	5
14	Blame myself for not knowing what to do	EO	1	2	3	4	5
15	Spend time with a special person	AO	1	2	3	4	5
16	Think about the event and learn from my mistakes	TO	1	2	3	4	5
17	Wish that I could change what had happened or how I felt	EO	1	2	3	4	5
18	Go out for a snack or meal	AO	1	2	3	4	5
19	Analyze my problem before reacting	TO	1	2	3	4	5
20	Focus on my general inadequacies	EO	1	2	3	4	5
21	Phone a friend	AO	1	2	3	4	5

Results and Comments

Overall, research using the CISS supports the notion that task-oriented coping is associated with greater wellbeing and positive adjustment to stress, while emotion-oriented and avoidance-oriented coping tend to be associated with greater distress and maladaptive adjustment. These associations have been found across a wide variety of stressful circumstances, including among people coping with academic worries (Zeidner, 1994), eating disorders (Koff & Sangani, 1998), chronic disease (Hurt et al., 2011), desire to quit smoking (Naquin & Gilbert, 1996), and insomnia (Morin, Rodrigue, & Ivers, 2003).
